# Whole-genome expression profile in zebrafish embryos after chronic exposure to morphine: identification of new genes associated with neuronal function and mu opioid receptor expression

**DOI:** 10.1186/1471-2164-15-874

**Published:** 2014-10-08

**Authors:** M Javier Herrero-Turrión, Iván Rodríguez-Martín, Roger López-Bellido, Raquel E Rodríguez

**Affiliations:** Instituto de Neurociencias de Castilla y León, University of Salamanca, Salamanca, 37007 Spain; Department of Basic Biomedical Sciences, European University of Madrid, Madrid, 28670 Spain; Department of Biochemistry and Molecular Biology, University of Salamanca, Salamanca, 37007 Spain; Instituto de Investigación Biomédica de Salamanca (IBSAL), Hospital Universitario de Salamanca - Edificio Virgen de la Vega, Salamanca, 37007 Spain; Instituto de Neurociencias de Castilla y León, Department of Biochemistry and Molecular Biology, University of Salamanca, C/ Pintor Fernando Gallego 1, Salamanca, 37007 Spain

**Keywords:** Morphine, Microarray, Zebrafish, Real-time quantitative PCR, Mu-opioid receptor, Gene expression, Addiction, Morpholino

## Abstract

**Background:**

A great number of studies have investigated changes induced by morphine exposure in gene expression using several experimental models. In this study, we examined gene expression changes during chronic exposure to morphine during maturation and differentiation of zebrafish CNS.

**Results:**

Microarray analysis showed 254 genes whose expression was identified as different by at least 1.3 fold change following chronic morphine exposure as compared to controls. Of these, several novel genes (*grb2, copb2, otpb, magi1b, grik-l, bnip4* and *sox19b*) have been detected for the first time in an experimental animal model treated with morphine. We have also identified a subset of genes (*dao.1, wls, bnip4* and *camk1γb*) differentially expressed by chronic morphine exposure whose expression is related to mu opioid receptor gene expression. Altered expression of *copb2, bnip4*, *sox19b, otpb*, *dao.1, grik-l* and *wls* is indicative of modified neuronal development, CNS patterning processes, differentiation and dopaminergic neurotransmission, serotonergic signaling pathway, and glutamatergic neurotransmission. The deregulation of *camk1γb* signaling genes suggests an activation of axonogenesis and dendritogenesis.

**Conclusions:**

Our study identified different functional classes of genes and individual candidates involved in the mechanisms underlying susceptibility to morphine actions related to CNS development. These results open new lines to study the treatment of pain and the molecular mechanisms involved in addiction. We also found a set of zebrafish-specific morphine-induced genes, which may be putative targets in human models for addiction and pain processes.

**Electronic supplementary material:**

The online version of this article (doi:10.1186/1471-2164-15-874) contains supplementary material, which is available to authorized users.

## Background

Morphine, the most widely known opioid agent, has been largely used for pain management. Besides non-analgesic uses of this drug, including experimental depression treatments as a control for opium addiction, has been developed [1, 2]. Despite these common uses, morphine produces disruptive negative secondary effects including sleepiness, drowsiness, blurred vision, constipation, and a decrease in blood pressure and appetite. With continuous use, morphine produces physical tolerance and addiction [1, 2]. At present, it is widely known that there are genetic components in the susceptibility to addiction [3, 4], but the specific genes that are involved in this process are scarcely known.

Opioids modify the expression profile of certain mRNAs in many tissues, including central nervous system (CNS), the main target of drugs of abuse [5]. With the use of microarray analysis, extensive studies have been focused on identifying morphine-induced changes in gene expression [6–19]. Other opioid agonist as oxycodone [20] and other drugs of abuse, such as cocaine, cannabis, methamphetamine, amphetamine, ecstasy, alcohol, heroin and nicotine [14, 21–25], have also been studied. Up to now, all microarray studies reporting gene expression changes in response to morphine have been conducted in rodents and humans. In particular, these studies were performed in whole tissues (mainly brain), distinct components of the nervous system (nucleus accumbens, striatum, hippocampus, frontal cortex, spinal cord), or cell cultures (primary and cell lines).

Zebrafish (*Danio rerio*) has been used in research related to drugs of abuse, studying the changes of gene expression produced by nicotine [26], amphetamine [27], and alcohol [28]. In addition, this teleost exhibits conditioned place preference (CPP), a technique used as a measure of drug reward or reinforcement [29], as responses to amphetamine and opiates like morphine [30–32]. These research works demonstrate the existence of a conserved drug-responsive ‘reward’ or reinforcement pathway in this teleost (as in all vertebrates), suggesting that this species may show adaptive changes and behavioural correlates of addiction after prolonged exposure to addictive drugs.

Zebrafish has also been used to study human disease-related pathways [33, 34], given this organism displays many benefits in comparison to other vertebrate animal models: small size, low cost, rapid development, transparency of the embryos, permeability to drugs, high fecundity, and transient genetic manipulation [35, 36]. In this sense, zebrafish is an important tool to analyze *in vivo* the molecular mechanisms related to the neurobiology of drug addiction, withdrawal and reward [31, 37–39] that cannot be fully established in other animals models. In contrast to mammalian embryos that develop in the uterus and are influenced by maternal biochemical processes, zebrafish embryos develop externally, avoiding the maternal effect on the embryos. This is essential when dealing with drug exposure, as the effects observed in mammalian embryos might be due to the susceptibility of the mother and not the embryo *per se*. The study of the direct effects of morphine in the embryos will provide a better understanding of the molecular mechanisms that underlie the physical and neurobehavioral defects shown in fetuses and offsprings after maternal morphine consumption [40, 41].

Therefore, we used microarray technology to obtain a profile of genes that are regulated by chronic morphine exposure on whole zebrafish embryos at 24 h post-fertilization (hpf), at the end of the segmentation period, during which the CNS is being formed and differentiated [37, 42, 43]. We have shown that zebrafish has endogenous opioids and opioid receptors with high homology with other species [44, 45]. The effects of morphine on embryos are probably mediated by the zebrafish mu opioid receptor (opioid receptor, mu1; *oprm1*), which exhibits highest affinity toward morphine from the known opioid receptors [46], and is the opioid receptor essential to mediate rewarding properties of most drugs of abuse [47, 48]. Our previous studies indicate that at 24 hpf, the expression of *oprm1* is higher than at other stages of development [49]. Therefore, the use of 24 hpf zebrafish embryos treated with morphine can provide information on the implication of the opioid system in the maturation and differentiation of CNS compared to any other stages of development. Our goal in this research was to differentiate functional classes of genes and individual candidates involved in the endogenous systems underlying susceptibility to morphine actions; hence, our efforts were focused on elucidating the functional significance of sets of differentially expressed genes related in some way to neuronal function and/or CNS development. To verify the results obtained by microarray, 12 selected genes were analyzed by quantitative reverse transcription real-time PCR (RT-qPCR). After “silencing” (knocking-down; KD) the *oprm1* gene expression by morpholino oligonucleotide injection, we identified a subset of genes that are regulated by morphine and are related to *oprm1* expression.

Our results show that morphine produces changes in gene expression in zebrafish embryos as has been obtained in rodents and humans [6–19]. Thus, our data, besides being analyzed independently, was also compared to previous studies, in an effort to determine which alterations in gene expression are species-specific (zebrafish *vs*. mammalians) and which may be common to all species in relation to addiction and pain studies. These results are important since several morphine-induced genes detected in zebrafish embryos may be putative targets in human models for addiction and pain processes.

## Results

### Microarrays analysis

**Figure 1:**
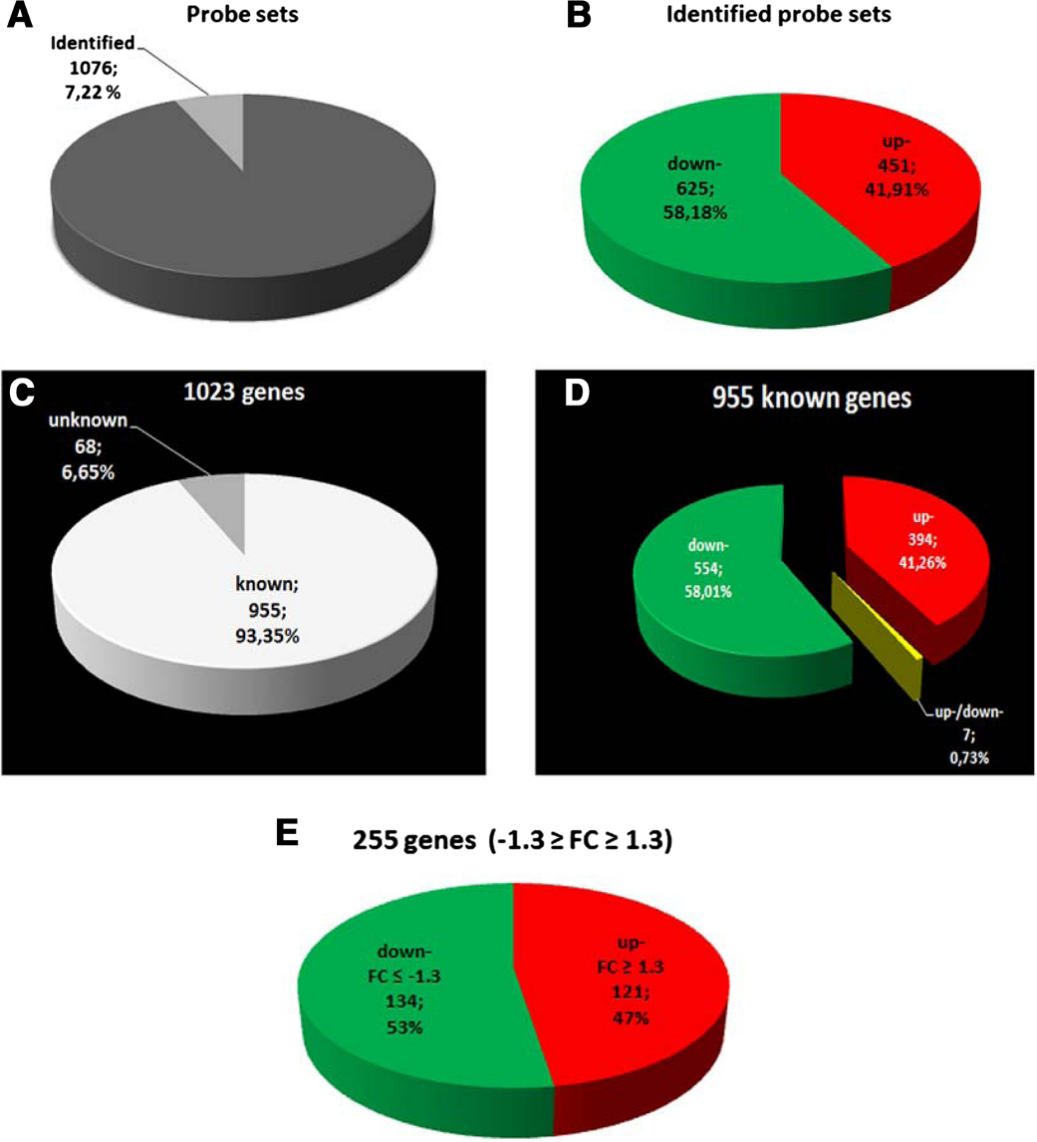
**Probe-sets and genes differentially expressed by chronic morphine treatment in zebrafish embryos.** Identified probe sets in comparison with total probes on the microarray **(A)** and detail of identified probe sets up- and down-regulated **(B)**. **(C)**: Number of identified genes (known and unknown). **(D)** Number of known genes and detail of genes up-, down- and up-/down-regulated. **(E)**: Number of identified genes with a fold change (FC) of at least 1.3. Numbers following the category names indicate the number of probe-set or genes. For the full list of probe-set/genes, see Additional file 1.

To examine the global transcriptional profiles of zebrafish embryos, RNAs generated from six pools of control zebrafish embryos and six pools of zebrafish embryos exposed to morphine were individually hybridized to Affymetrix Zebrafish Genome Arrays. A set of gene products corresponding to a total of approximately 14900 probes on the arrays were confidently detected, based on signal intensity at a fixed value above background levels. Differentially expressed genes were identified through the Significance Analysis of Microarrays (SAM) method. As shown in Figure 1, we identified a total of 1023 gene expression changes in the above mentioned comparison (representing 1076 probe sets; Figure 1A-C). Of these, 955 genes were identified as known genes; 401 genes were significantly up-regulated and 561 down-regulated (Figure 1C-D). Moreover, as shown in Figure 1D, seven genes regulated, after chronic exposure to morphine, are represented by more than one probe set in the zebrafish microarray, and each of the probe set is differently deregulated (up- or down-regulated); for example: acireductone dioxygenase 1 (*adi1*; Dr.20538.1.A1, +1.15 fold change (FC) and Dr.9210.2.S1, −1.23 FC) and selenoprotein X, 1a (*sepx1a*; Dr.147.1.A1, +1.43 FC; Dr.24956.1.S1, −1.27 FC; and Dr.16417.1.A1; −1.46 FC). Therefore, distinct gene expression changes obtained for each one of the probe sets of each gene might mean that these genes show alternative splicing and each one of them has at least two isoforms that are differently regulated by exposure to morphine. Finally, we specifically identified 255 genes (representing 265 probe sets) showing differential expression with a FC of at least 1.3: 121 genes (126 probe sets) were up-regulated and 134 genes (140 probe sets) were down-regulated by chronic morphine treatment (Figure 1E; see complete list of differentially expressed genes in Additional file 1).

### Gene ontology analysis

**Figure 2:**
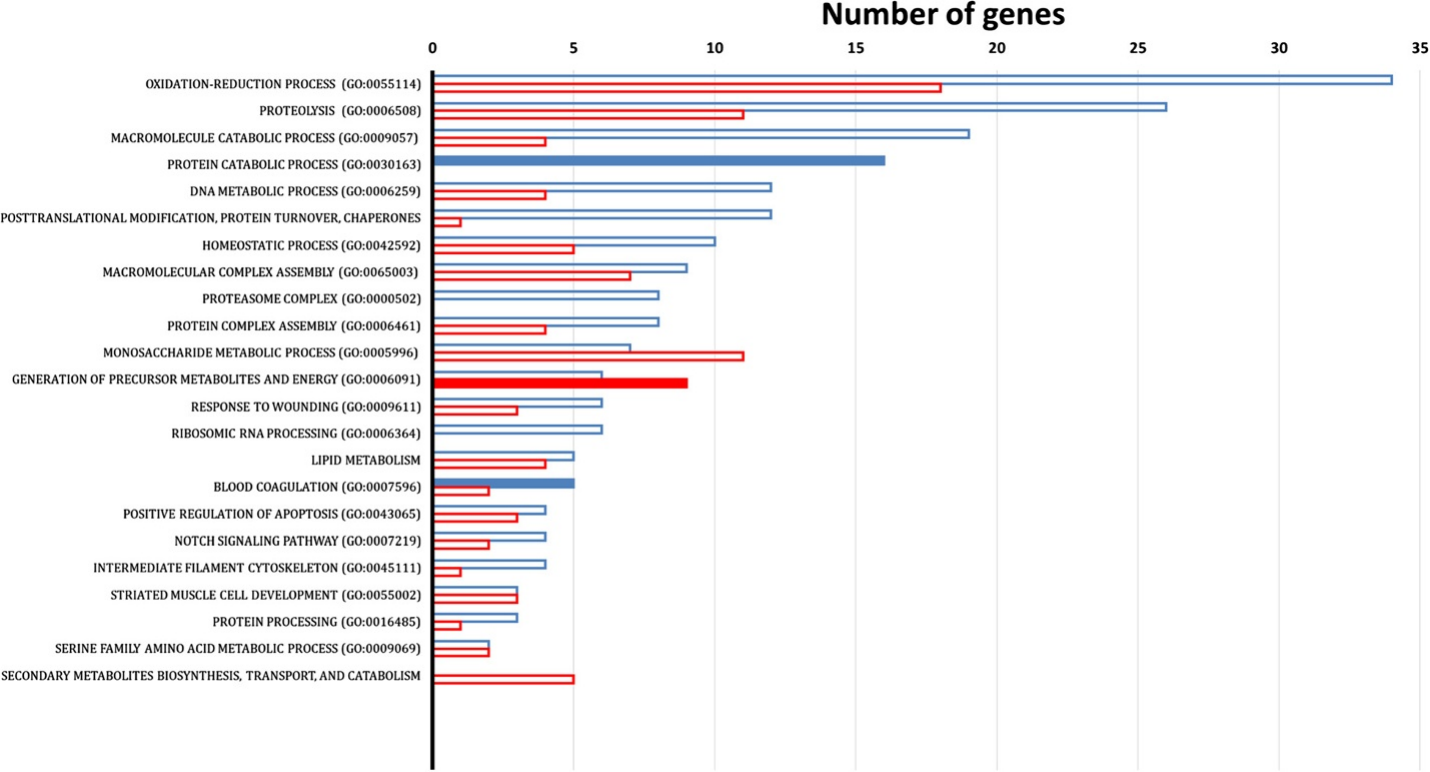
**Gene ontology analysis of zebrafish embryos gene collection.** Genes were categorized with the Biological Process domain. Significantly enriched GO terms have a probability lower than 0.01 (*P* value) and include at least three genes. GO terms are shown for both up- (in red) and down-regulated (in blue) groups if they are significantly enriched in at least one of them. Significantly enriched GO terms are indicated as full bars whereas non-significantly enriched terms are displayed as empty bars. Bars represent the number of genes assigned with the corresponding GO term. For the full list of represent genes in each category, see Additional file 3.

To enhance biological interpretation of the differentially expressed genes from our microarray studies, we sought if any of the biological processes or molecular functions were over-represented by these genes. Therefore we performed a function enrichment analyses for the differentially expressed genes using the functional classification tool *DAVID Bioinformatics resources*. A representative selection of meaningful categories is illustrated in Figure 2 and Table 1. This analysis shows that some categories are specifically enriched in either up- or down-regulated genes, whereas other categories show both types of regulation. Genes involved in oxidation-reduction processes (Gene Ontology (GO):0055114), proteolysis (GO:0006508), monosaccharide metabolic processes (GO:0005996), macromolecular complex assembly (GO:0065003) and generation of precursor metabolites and energy (GO:0006091) represent an important part of both up- and down-regulated genes. Macromolecule catabolic processes (GO:0009057), DNA metabolic process (GO:0006259), protein catabolic process (GO:0030163), homeostatic process (GO:0042592), protein complex assembly (GO:0006461), proteasome complex (GO:0000502), and in general, post-translational modification, protein turnover, chaperones, mainly include down-regulated genes. There is an over-representation of up-regulated transcripts associated with secondary metabolites biosynthesis, transport, and catabolism (for complete details, see supplementary Additional files 2 and 3).

**Table 1:** **Examples of significantly enriched GO annotation for the list of morphine-responsive genes**

Enriched GO annotation	De‐regulation		Up‐regulation			Down‐regulation		
	No. genes	P value	No. genes	P value	Genes	No. genes	P value	Genes
**Oxidation‐reduction process (GO:0055114)**	52	3.4E + 10	18	0.013	acox1, cyp11a1, cyp26b1, cyp27a1.4, d2hgdh, gapdhs,	34	3.7E + 10	adh8b, aldh18a1, cp, cyp2aa4, dao.1, dhfr, dhrs13b, mdh1b, phyhd1, ptgr1, sepx1a
**Proteolysis (GO:0006508)**	37	0.049	11		ube2d2	27	0.036	f7, rad23b, spcs2, uchl5, vsg1
**Macromolecule catabolic process (GO:0009057)**	23	0.003	4		ube2d2	19	4.3E + 12	ctbs. LOC553461, rad23b, uvsg1
**Monosaccharide metabolic process (GO:0005996)**	18	6.8E + 08	11	3.8E + 09	aldocb,galk2, gapdhs, gpia	7	0.050	mdh1b, pklr
**DNA metabolic process (GO:0006259)**	17	0.080	5		mlf1, mphosph8	12	0.054	orc4, rad23b
**Protein catabolic process (GO:0030163)**	16	0.002				16	0.002	LOC553461, rad23b, uchl5, vsg1
**Macromolecular complex assembly (GO:0065003)**	16	0.003	7	0.044	h1fx	9	0.065	fgg, tubb5
**Generation of precursor metabolites and energy (GO:0006091)**	15	0.012	9	0.006	aldocb, gapdhs, gpia	6		mdh1b, pklr
**Homeostatic process (GO:0042592)**	15	0.025	5		fth1	10	0.060	
**Posttranslational modification. protein turnover. chaperones**	13	0.005	1			12	1.9E + 12	
**Protein complex assembly (GO:0006461)**	12	0.005	4			8	0.024	fgg, tubb5
**Lipid metabolism**	9	0.005	4	0.087	acsl1, acsl5	5	0.072	
**Response to wounding (GO:0009611)**	9	0.010	3		cxcl12a	6	0.042	f7, fgg
**Proteasome complex (GO:0000502)**	8	5.1E + 11				8	5.1E + 11	
**Blood coagulation (GO:0007596)**	7	7.1E + 11	2		tfpia	5	0.005	f7,fgg
**Positive regulation of apoptosis (GO:0043065)**	7	0.010	3		bnip4	4		
**Striated muscle cell development (GO:0055002)**	6	0.060	3		cxcl12a	3		tnnt2a
**Intermediate filament cytoskeleton (GO:0045111)**	5	0.043	1		cyp27a1.4	4	0.043	krt23
**Notch signaling pathway (GO:0007219)**	5	0.072	2		dlc	3		vsg1
**ribosomic RNA processing (GO:0006364)**	5	0.086				5	0.021	
**Secondary metabolites biosynthesis. transport. and catabolism**	5	0.044	5	0.044	cyp11a1. cyp26b1. cyp51			
**protein processing (GO:0016485)**	4	0.036	1			3		spcs2
**serine family amino acid metabolic process (GO:0009069)**	4	0.065	2		gcshb	2		dhfr

The complete results of the Gene Ontology (GO) analysis are presented in Additional file 3.

**Figure 3:**
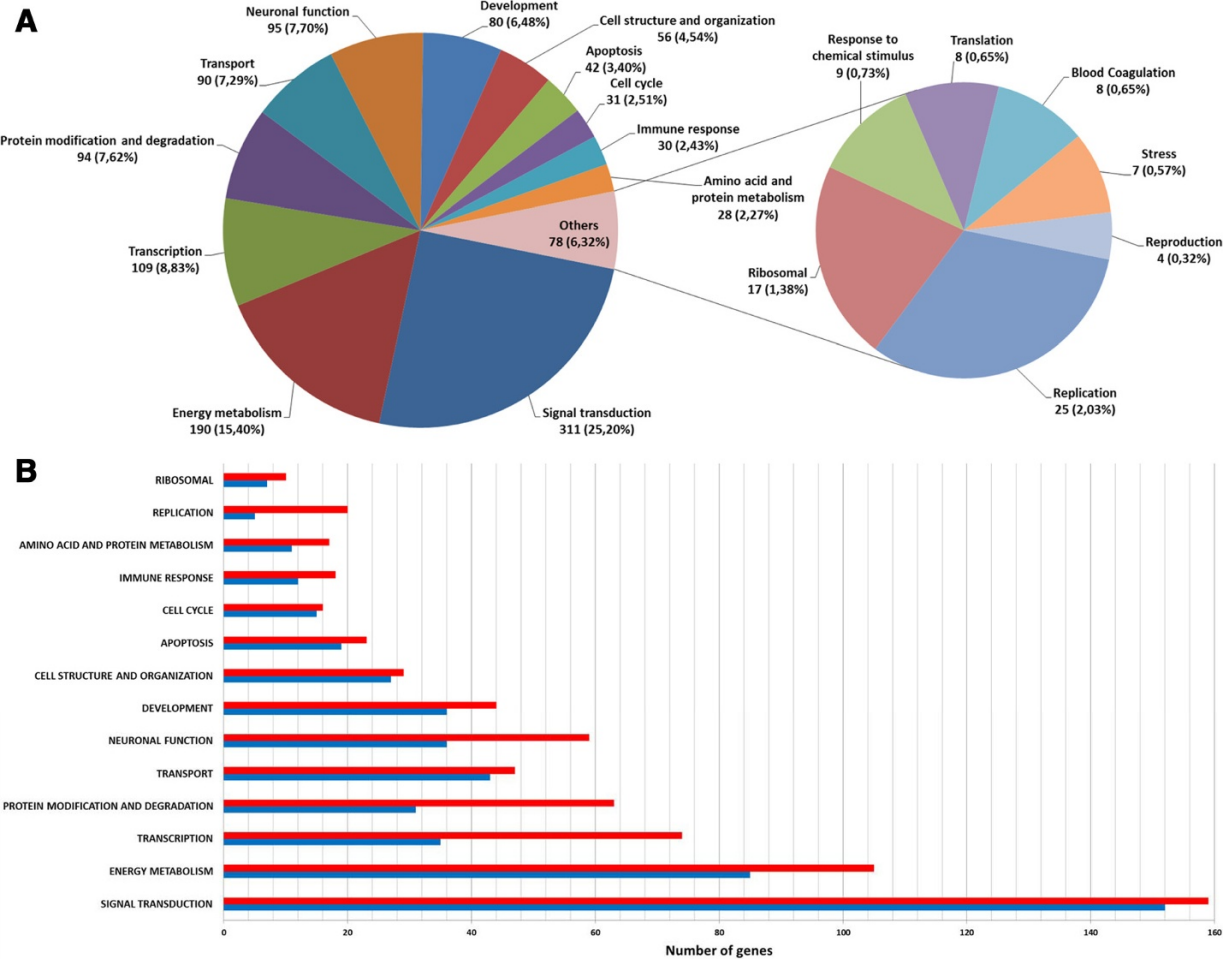
**Functional categories and number of genes found to be modified by morphine in each category. (A)** Functional categories of genes found to be associated with the exposure to morphine in zebrafish embryos. Numbers following the category names indicate the number of genes in the category, and those in parentheses are the corresponding percentages. **(B)** Number of genes (up-, in blue, and down-regulated, in red) by functional category. For the full list of genes, see Table 2 and Additional file 4.

**Figure 4:**
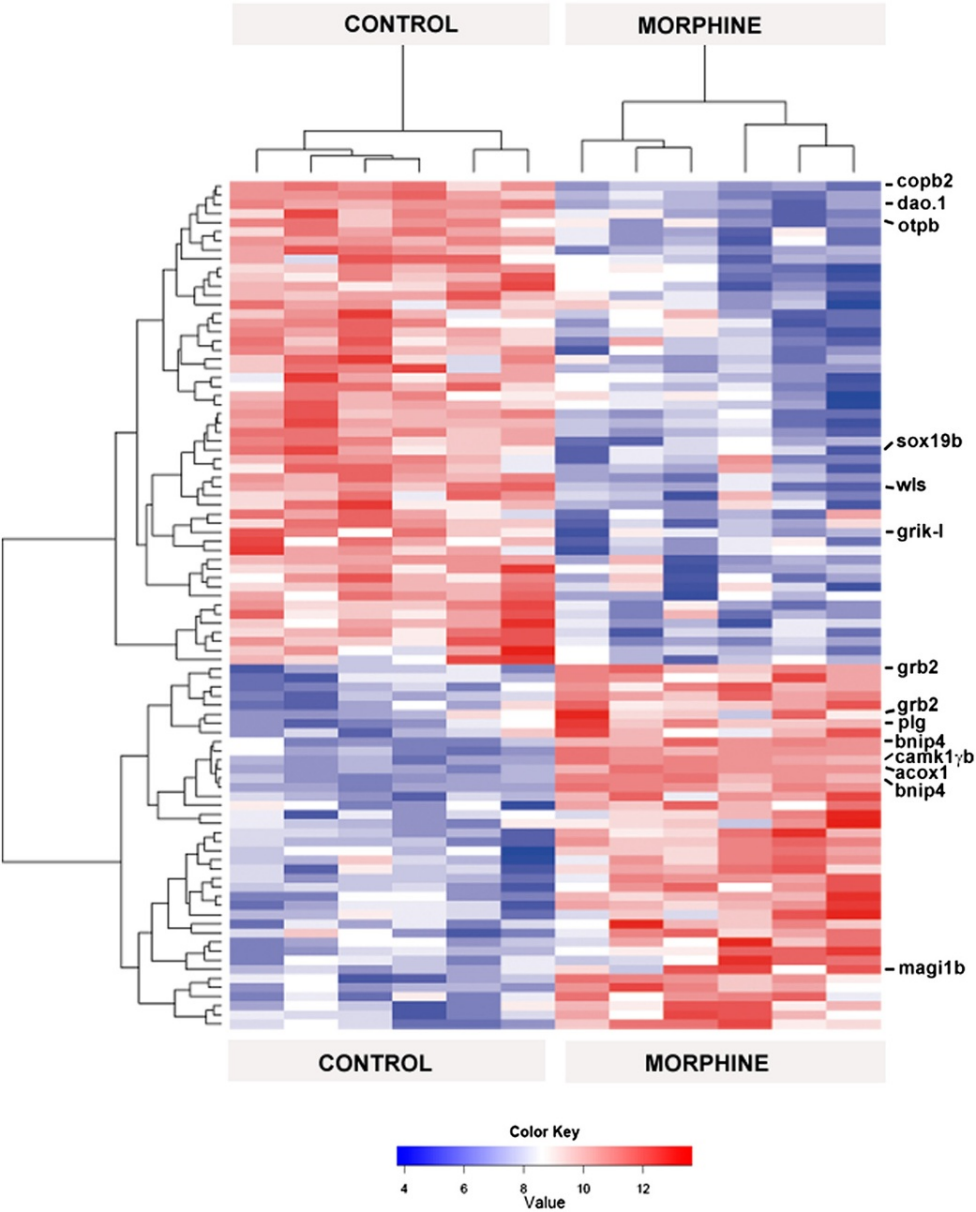
**Hierarchical cluster of probe sets regulated in zebrafish embryos following chronic morphine exposure.** Raw data from six independent hybridizations are presented for the two experimental groups: control and morphine-treated embryos zebrafish. The probe-set selection was based on a standard statistical analysis with Robust Multi-array Analysis (see Methods for details). Hierarchical cluster analysis was performed using Cluster 3.0 and Treeview software and the tree image represents low (in blue) or high expression (in red) of probe sets. These probe sets represent 87 genes (representing 93 probe sets) involved in Neuronal Function (a complete list is provided in Additional file 4). The twelve genes (fourteen probe sets) chosen for validation of microarray data by RT-qPCR are listed (right hand side of the figure).

For our study, the different categories within Gene Ontology Biological Process can be classified in other “functional” categories according to databases and consulted publications (see Discussion section). As shown in Figure 3, more than one fourth of differentially expressed genes are related to signal transduction and other biological processes, including energy metabolism, transcription, protein modification and degradation, neuronal function, transport, development, cell structure and organization, apoptosis, amino acid and protein metabolism, replication and stress (see Table 2, and for complete list also see supplementary Additional file 4). In particular, the genes related to neuronal function are shown in a hierarchical cluster in Figure 4 and listed in Table 3. These genes were further investigated in this study resulting in the following categories of GO Biological Process (Additional file 4): regulation of transcription [GO:0045449; as for example, cAMP responsive element binding protein 3-like 3 (*creb3l3*), developing brain homeobox 1b (*dbx1b*), distal-less homeobox protein 5a (*dlx5a*), c-fos FBJ murine osteosarcoma viral oncogene (c-*fos*), hairy-related 4.2-like (*her4.2*), jun B proto-oncogene and b (*juna* and *junb*), neurogenic differentiation factor 6-B (*neurod6b*), orthopedia b (*otpb*), SRY-box containing gene 19b (*sox19b*), and *sox21b*], neuron differentiation [GO:0030182; v-erb-b2 erythroblastic leukemia viral oncogene homolog 2 (*erbb2*), *otpb*, N-ethylmaleimide-sensitive factor a (*nsfa*), delta-like protein C Precursor (*dlc*)], somitogenesis (GO:0001756; *dlb, dlc, her4.2*) and glutamine biosynthetic process [GO:0006542; solute carrier family 1 (glutamate/neutral amino acid transporter), member 4 (*slc1a4*) and solute carrier family 1 (glial high affinity glutamate transporter), member 3a (*slc1a3a*)].

**Table 2:** **Functional categories and number of genes found to be modified by morphine in each category**

Functional categories	N genes	Up‐regulation		Down‐regulation	
**Signal transduction**	311	152	ppp1r14ab, camk1γb, grb2, sept9a, itih3, ahnak, hrc, sesn3, megf6, btr22, tmem101, magi1b, vldlr*, hspb1*, plg*	159	LOC566752, a2ml, sh3bp5la, fam115, txndc9, tctex1d2, rcan2, rnf220b, hspb8, slc1a4*, wls*, grik‐l*
**Energy metabolism**	190	85	itih3, acox1, soat1, cyp17a2, cyp51, cox4.2, alpp, aldocb, pcmt*, plg*	105	aldh8a1, pklr, mpdu1b, mdh1b, phyhd1, hrasls, glb1l, glud1b*
**Transcription**	109	35	dcps, zbtb16, creb3l3*, junb*, dlx5a*, fos*, dlx4a*	74	sox19b, mettl11a, noc2l, dbx1b, junbb, her4.2, otpb*, sox11b*, sox21b*, hdac6*, dnmt3b*, sox19a*, sox11b*, mir429b
**Protein modification and degradation**	94	31	ube2d2, hspb1*, plg*	63	pcmt, rad23b, fbxo25, hspb8
**Transport**	90	43	slc31a1, abca12, apoea, abcb5, ctssb.2*, slc3a2*, abt4*, slc1a3a*, slc7a8*	47	a2ml, bcap31, aqp12, aqp3a, vamp5, slc1a4*, vamp4*, hiat1b*, apoIV*, brp44*, kctd12.2*
**Neuronal function**	95	36	bnip4, ppp1r14ab, acox1, serinc5, maptb, magi1b, hspb1*, plg*, aplp*, slc1a3a*, creb3l3*, hsp90a.1*, junba*, dlx5a*, fos*, glula	59	pcmt, dao.1, sox19b, a2ml, mettl11a, astn1, noc2l, copb2, dbx1b, junbb, her4.2, slc1a4*, her3*, wls*, grik‐like*, otpb*, cyrano*, sox11b*, sr*, sox21b*, glulb*, glud1b*, dnmt3b*, kctd12.2*, sox11b*, mir429b*
**Development**	80	36	bnip4, ppp1r14ab, camk1γb, acox1, ahnak, maptb, zbtb16, cyp11a1, magi1b, hspb1*, plg*, dlx5a*, dlx4a*	44	sox19b, a2ml, hspb8, noc2l, copb2, dbx1b, her4.2, her3*, wls*, otpb*, cyrano*, sox11b*, sox21b*, hoxb8a*, dnmt3b*, kctd12.2*, sox11b*
**Cell structure and organization**	56	27	mybpc2b, maptb, col5a1	29	mylz2, krt1‐19d
**Apoptosis**	42	19	bnip4, hspb1*, bbc3*	23	hspb8, noc2l, bcap31, casp9*, mir429b*
**Cell cycle**	31	15	sept9a, sesn3, ccnt1, hsp90a.1*, ccnd1*	16	ccnb3*
**Immune response**	30	12	ccl‐c11b, cxcl12a, ctssb.2*, plg*	18	crfb6
**Amino acid and protein metabolism**	28	11	hnmt, eif4a2, eif3hb, eif2c2, slc3a2*, slc7a8*	17	fah, dao.1, aldh18a1, iars, eif4g2b*, sr*, eif4g2a*, eif2s1l*
**Replication**	25	5	h1fx	20	rad23b, mettl11a, noc2l, h2afx*, dnmt3b*
**Ribosomal**	17	7	rpf2	10	nob1, fcf1
**Response to chemical stimulus**	9	3	hspb1*, hsp90a.1*	6	hspb8
**Translation**	8	2	gcn1l1	6	hbs1l*
**Blood Coagulation**	8	3	serping1	5	fgg
**Stress**	7	3	sesn3, hspb1*, hsp90a.1*	4	hspb8
**Reproduction**	4	3		1	

Some up-regulated genes with FC > 1.0 and down-regulated genes FC < −1.0 are marked with an asterisk (*).

**Table 3:** **Genes involved in neuronal function with −1.30 ≥ R fold ≥ 1.30**

Gene symbol	Gene description	R.fold	Probeset ID
***UP‐REGULATION***			
bnip4	BCL2/adenovirus E1B interacting protein 4	2,357	Dr.24320.1.S1_at
ppp1r14ab	Protein phosphatase 1, regulatory (inhibitor) subunit 14Ab	2,239	Dr.19567.1.S1_at
camk1γb	Calcium/calmodulin‐dependent protein kinase Iγb	2,164	Dr.9852.1.A1_at
acox1	Acyl‐Coenzyme A oxidase 1, palmitoyl	1,741	Dr.3576.1.A1_at
serinc5	Serine incorporator 5	1,480	Dr.7467.1.S1_at
maptb	Microtubule‐associated protein tau b	1,462	Dr.16118.1.A1_at
dlc	Delta‐like protein C Precursor	1,375	Dr.16183.1.S1_at
dmxl2	Dmx‐like 2 (rabconnectin‐3)	1,367	Dr.12467.1.A1_at
lmbr1l	limb region 1 like	1,346	Dr.19947.1.S1_at
fam120c	Family with sequence similarity 120C (constitutive coactivator of PPAR‐gamma‐like protein 2)	1,334	Dr.18209.1.A1_at
magi1b	Membrane associated guanylate kinase, WW and PDZ domain containing 1b (BAI1‐associated protein 1)	1,300	Dr.4477.1.A1_at
***DOWN‐REGULATION***			
dao.1	D‐amino‐acid oxidase 1	−1,943	Dr.3663.1.A1_at
sox19b	SRY‐box containing gene 19b	−1,802	Dr.25405.1.A1_at
a2ml	Alpha‐2‐macroglobulin protein‐like	−1,801	Dr.3025.1.A1_at
astn1	Astrotactin 1	−1,484	Dr.14729.2.A1_at
enc1	Ectodermal‐neural cortex (with BTB‐like domain)	−1,466	Dr.9565.1.S1_at
copb2	Coatomer protein complex, subunit beta 2	−1,393	Dr.14687.1.A1_at
mchr1b	Concentrating hormone receptor 1b	−1,387	Dr.24966.1.S1_at
dbx1b	Developing brain homeobox 1b	−1,364	Dr.8072.1.S1_at
junbb	Jun B proto‐oncogene b	−1,356	Dr.737.1.A1_at
her4.2	Hairy‐related 4.2	−1,324	Dr.20386.1.S1_at
lrrn1	Leucine rich repeat neuronal 1	−1,309	Dr.24292.3.A1_a_at

Genes were identified as being differentially regulated (up- and down-regulated) in embryos zebrafish by morphine treatment.

### Validation of microarray data by quantitative reverse transcription real-time PCR (RT-qPCR)

RT-qPCR analysis was used to confirm a set of gene expression changes observed in the microarray analysis. Chosen genes for RT-qPCR confirmation were mainly selected based on ontological categories with potential roles in the nervous system.

To choose the most stable genes as internal references for RT-qPCR data normalization, four candidates [*β*-actin (*β-act*), ribosomal protein L13a (*rpl13a*), *β*-2-microglobulin (*β2 mg*) and elongation factor-1α (*ef1*α)] were selected according to their expression levels detected in the microarray studied. The expression of these four genes was also measured by RT-qPCR. The *NormFinder software* [50] was used to calculate the intra- and inter-group variations in their expression. Our results indicate that *β-act* is the most stable gene, whereas *rpl13a*, *β2 mg* and *ef1*α are less stable (data not shown). Thus, the mean of threshold cycle (Ct) value and primer efficiency value of *β-act* was used for normalization.

As shown in Table 4 and Figure 4, we examined a total of 12 regulated genes after chronic morphine exposure on zebrafish embryos (represented by 14 probe sets in the microarray system studied) using RT-qPCR technique. The up-regulated genes include: acyl-Coenzyme A oxidase 1, palmitoyl (*acox1*), growth factor receptor-bound protein 2 (*grb2*), and Ca^2+^/calmodulin-dependent protein kinase Iγb (*camk1γb*). The group of down-regulated genes is formed by the transcription factor *sox19b* (also named *sox31*) and wntless (*wls*, also known as *gpr177*).

**Table 4:** **Comparison between microarray and RT-qPCR data for selected genes**

Transcripts cluster Id	Gene names	Microarray data R.fold	RT‐qPCR data	
			Fold change	***P***‐value
**Up‐regulation**				
Dr.24320.1.S1_at Dr.21771.1.S1_at	*bnip4*	2.36/1.23	1.90 ± 0.41*	7.42 E‐05
Dr.9852.1.A1_at	*camk1γb*	2.16	1.90 ± 0.57*	1.41 E‐05
Dr.7717.2.A1_at Dr.7717.1.A1_at	*grb2*	2.02/1.58	1.27 ± 0.15*	8.98 E‐04
Dr.3576.1.A1_at	*acox1*	1.74	2.03 ± 0.43*	2.04 E‐05
Dr.4477.1.A1_at	*magi1b*	1.30	1.55 ± 0.12*	5.43 E‐05
Dr.3645.1.S1_at	*plg*	1.26	−1.06 ± 0.21	0.299
***Down‐regulation***				
Dr.3663.1.A1_at	*dao.1*	−1.94	−3.31 ± 0.11*	3.59 E‐08
Dr.25405.1.A1_at	*sox19b*	−1.80	−2.09 ± 0.19*	0.001
Dr.14687.1.A1_at	*copb2*	−1.39	−1.69 ± 0.19*	0.004
Dr.3546.1.S1_at	*wls*	−1.25	−1.67 ± 0.16*	0.006
Dr.3211.1.A1_at	*grik‐l*	−1.23	−2.94 ± 0.21*	2.53 E‐04
Dr.8118.1.A1_at	*otpb*	−1.22	−1.64 ± 0.23*	0.004

Values of fold change by RT-qPCR are given as mean of fold change ± standard deviation. The study includes samples used for microarray hybridization and samples from independent treatments; a total of n = 8. For better comparison of the results obtained with both techniques, microarray and RT-qPCR, Morphine *vs*. Control fold changes are indicated with positive and negative values for up- and down-regulations, respectively. A one-way *t*-test was performed to determine whether fold changes obtained for morphine-regulated genes were different from 1 and significant values are indicated with asterisks.

It is of interest that the probe set that presents the highest gene expression (Dr.26538.1.A1; +4.73 FC), putatively identified by Affymetrix as wls, do not aligned to *wls* genomic locus. Our *in silico* analyses demonstrated that when performing a Blastn in the Ensembl site, using as query an unique sequence of 59 nucleotides formed by the overlapping of 16 probes which constitute this probe set, the query sequence is aligned in two unidentified non-coding regions (on chromosomes 6 and 8) that do not belong to *wls* genomic locus located on chromosome 2. Therefore, Dr.26538.1.A1 probe set cannot be identified as *wls* gene (see other genes differentially expressed by morphine validation by our in silico studies in Additional file 5). Concerning the *wls* gene, we have detected that uniquely the Dr.3546.1.S1 probe set is complementary to this gene (Table 4).

We also analyzed four genes regulated by chronic morphine treatment involved specifically in dopaminergic and serotonergic neurotransmission. A gene putatively related to serotonergic neurotransmission is BCL2/adenovirus E1B interacting protein 4 (*bnip4*). Three genes involved in dopaminergic neurotransmission are the plasminogen (*plg*), coatomer protein complex subunit beta 2 (*copb2*), and the transcription factor *otpb*. Furthermore, we included in our study three genes related to glutamatergic neurotransmission: one up-regulated (membrane associated guanylate kinase, WW and PDZ domain containing 1b, *magi1b*) and two down-regulated (D-amino-acid oxidase 1, *dao.1*, and a kainate-like receptor, *grik-l*).

We showed that two regulated genes, after chronic morphine exposure, are represented by more than one probe set in the zebrafish microarray (*bnip4* and *grb2*; Table 4). Distinct gene expression changes obtained for each one of the probe sets for each gene might mean that these genes show alternative splicing and each one of them has at least two isoforms that are differently regulated by exposure to morphine.

All genes chosen for RT-qPCR confirmation and involved in neuronal functions are also related to other functional categories according to databases and consulted publications, such as development (*acox1, camk1γb, sox19b, plg, otpb, copb2, wls*, *bnip4* and *magi1b*), signal transduction (*camk1γb, grb2, plg, wls, grik-l* and *magi1b*), transcription (*sox19b* and *otpb*), apoptosis (*bnip4*), energy metabolism (*acox1* and *plg*) and, specifically, amino acid and protein metabolism (*dao.1*) (see details in Table 2). It should be emphasized that among all genes confirmed by RT-qPCR some have previously been identified in distinct studies with mammals, such as *Camk1γ, Plg, Dao* and *Wls* [10, 12, 15, 51, 52] (Table 5).

**Table 5:** **Example of genes differentially expressed by morphine in our study and identified in other species**

Probeset ID	Genename	Description/validation in silico	R.fold	Species	Reference
***Up‐regulation***					
Dr.9852.1.A1_at	camk1γb	Calcium/calmodulin‐dependent protein kinase Igb	2,16	Mouse	[12]
Dr.23925.1.A1_at	soat1	Sterol O‐acyltransferase (acyl‐Coenzyme A: cholesterol acyltransferase) 1	1,69	Rat	[16]
Dr.19223.1.S2_at	aldocb	Aldolase C, fructose‐bisphosphate, b	1,49	Rat	[105]
Dr.11457.1.S1_at	zbtb16	Zinc finger and BTB domain containing 16	1,44	Mouse	[12, 65]
Dr.12489.1.S1_at	mlf1	Myeloid leukemia factor 1	1,44	Mouse	[8]
Dr.18505.1.S1_at	ccnt1	Cyclin‐T1	1,44	Mouse	[6]
Dr.822.1.S3_at	cxcl12a	Chemokine (C‐X‐C motif) ligand 12a (stromal cell‐derived factor 1)	1,38	Mouse	[8]
Dr.23722.1.S1_at	cyp27a1.4	Cytochrome P450, family 27, subfamily A, polypeptide 1, gene 4	1,35	Mouse	[8]
Dr.9423.1.S1_at	ndufb2	NADH dehydrogenase (ubiquinone) 1 beta subcomplex, 2, 8 kDa	1,31	Mouse	[6]
Dr.867.1.S1_at	wdr1	WD repeat domain 1	1,28	Rat	[105]
Dr.12378.1.S1_at	hspb1	Heat shock protein, alpha‐crystallin‐related, 1	1,27	Rat & mouse	[16, 55]
Dr.3645.1.S1_at	plg	Plasminogen	1,26	Rat & mouse	[12, 15]
***Down‐regulation***					
Dr.3663.1.A1_at	dao.1	D‐amino‐acid oxidase 1	−1,94	Rat	[51, 52]
Dr.9860.1.S1_at	mdh1b	Malate dehydrogenase 1b, NAD (soluble)	−1,79	Rat	[105]
Dr.19735.1.S1_at	phyhd1	Phytanoyl‐CoA dioxygenase domain containing 1	−1,56	Mouse	[55]
Dr.5687.1.A1_at	hspb8	Heat shock protein, alpha‐crystallin‐related, b8	−1,45	Mouse	[8, 55]
Dr.737.1.A1_at	junbb	Jun B proto‐oncogene b	−1,36	Rat & mouse	[7]
Dr.3546.1.S1_at	wls	wntless homolog (Drosophila)	−1,25	Mouse	[10]

**Figure 5:**
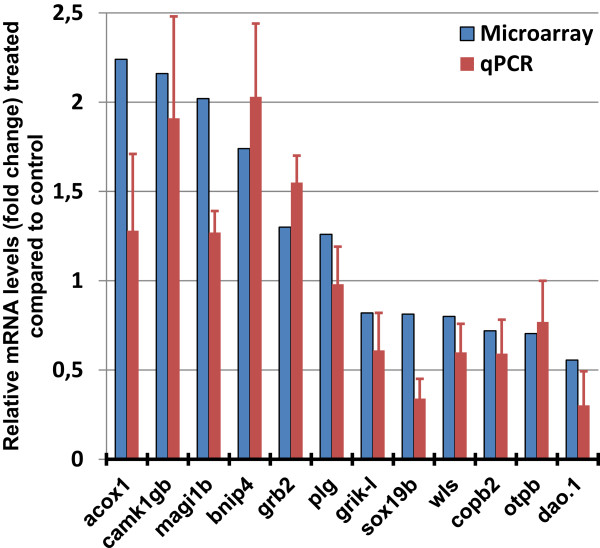
**Quantitative real-time PCR (RT-qPCR) was used to validate the microarray data.** The genes selected showed similar expression changes when assessed by RT-qPCR as determined by microarray analysis. Data are presented as morphine *vs.* control fold changes.

For our RT-qPCR analysis, we used the same RNA samples as those used for the microarray hybridization experiment, and additionally, samples from eight independent chronic morphine treatments were also used. Results from all the RT-qPCR experiments are summarized in Table 4 and Figure 5: *acox1, bnip4, camk1γb, magi1b*, and *grb2* appeared as the most up-regulated transcripts with 2.03 ± 0.43, 1.90 ± 0.41, 1.90 ± 0.57, 1.55 ± 0.12, and 1.27 ± 0.15 FC, respectively. We also clearly confirmed down-regulation of *sox19b*, *grik-l*, *dao.1*, *copb2*, *wls* and *otpb* transcripts (−3.31 ± 0.11, −2.94 ± 0.21, −2.09 ± 0.19, −1.69 ± 0.19, −1.67 ± 0.16, −1.64 ± 0.23 FC, respectively). However, the up-regulation of *plg* was not confirmed by RT-qPCR since the result showed no significant deregulation. Differences between RT-qPCR and microarray experiments occur for several reasons, including the fact that different probes are used for the microarray and RT-qPCR experiments (which can capture differential expression in splice variants), differences in the methods for normalization of expression data and possible false-positive expression changes. In addition, lower correlations between RT-qPCR and microarray results, such as for *plg* gene (+1.26 FC), were consistently reported for genes exhibiting small degrees of changes, generally less than 1.4 FC [53].

**Figure 6:**
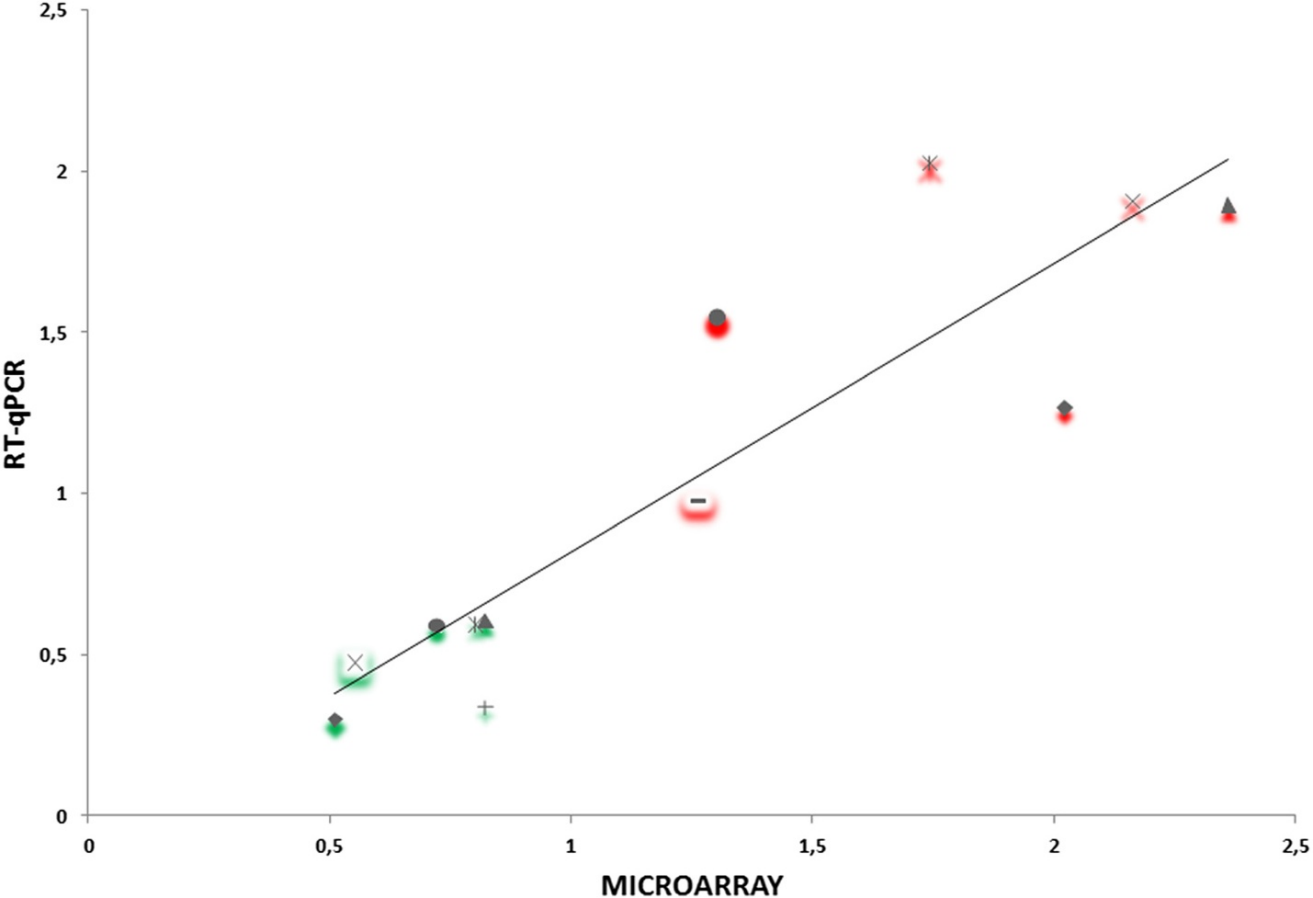
**Validation of microarray data by RT-qPCR.** Correlation between microarray (x-axis) and RT-qPCR (y-axis) data. This correlation was analyzed by Spearman’s rho test and a high statistical significance [r (12) = 0.884, p < 0.001] was observed. Values used in this graph were taken from results presented in Table 4. Each gene is labeled with a symbol and up- and down-regulated genes are marked in red and green, respectively: ♦, *grb2* and *dao.1*; ●, *magi1b* and *copb2*; ▲, *bnip4* and *otpb*; x, *camk1gb* and *sox19b*; Ӿ, *acox1* and *wls*; +, *grik-l*; −, *plg*. In the case of two regulated genes (*bnip4* and *grb2*) after chronic morphine exposure that are represented by two probe sets each one, we have included in this figure the highest microarray data: Dr. 24320.1, *bnip4* + 2.36 FC (▲), and Dr. 7717.2, *grb2*, +2.02 FC (♦).

Correlation between the microarray and RT-qPCR results for the 12 selected genes was then performed and the statistical significance of the correlation determined. As Morey *et al.* [53] suggest, prior to performing correlation analysis, the dataset should be tested for normality using the Shapiro-Wilk test; our data points were not normally distributed and therefore Spearman’s rho test was used. This test is the rank-based non-parametric equivalent of the more commonly used Pearson’s correlation calculation [53]. The expression data for this gene set detected by microarray and RT-qPCR are plotted in Figure 6. The RT-qPCR and microarray methods showed excellent qualitative agreement on both up- and down-regulated genes. The correlation between microarray and RT-qPCR data obtained a high statistical significance [r (12) = 0.884, *p* < 0.001]. As mentioned before, the expression of several morphine-induced genes identified in previous studies (see Table 5), such as *camk1γb*, *dao.1* and *wls*, was also analyzed to evaluate the data reliability and the sensitivity of microarray analysis. As shown in Figure 5, the induced expression of *camk1γb* and decreased expression of *wls* was successfully detected by both RT-qPCR and microarray approaches. These results confirmed the reliability of microarray analysis method in this study.

### The role of *oprm1*gene in morphine-induced regulation of differentially expressed genes in zebrafish embryos

**Figure 7:**
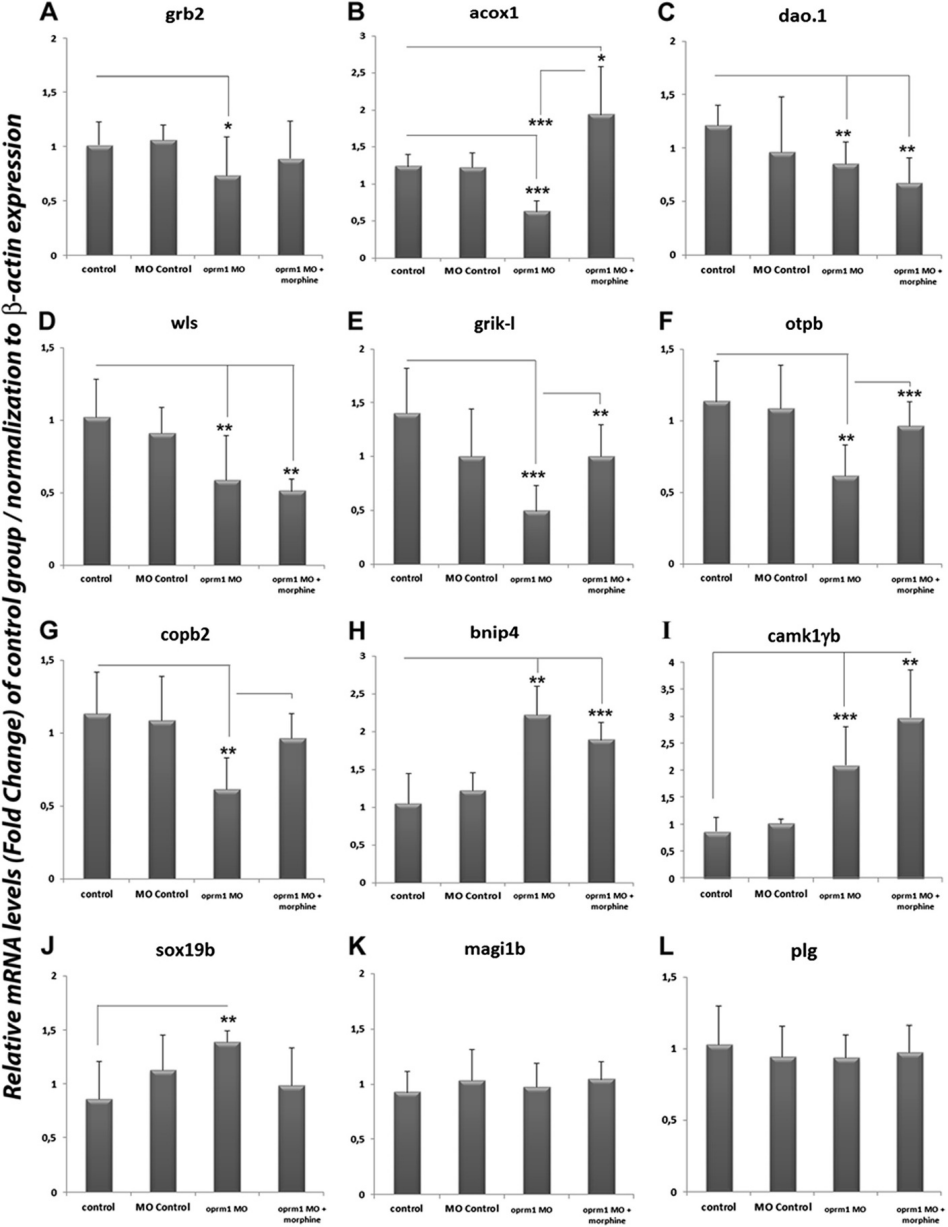
**Knockdown of μ opioid receptor (** ***oprm1*** **).** Expression levels of *grb2, acox1, dao.1, wls, grik-l, otpb, copb2, bnip4, camk1gb, sox19b, magi1b* and *plg* of the Control group, the Morpholine (MO) control, Morpholine *oprm1* (*oprm1*-MO) and *oprm1*-MO exposure to morphine (*oprm1*-MO + morphine). The expression levels were quantified using RT-qPCR analysis and were normalized to *β-actin* expression. Each bar represents the FC ± SD (*n* = 8). Data were analyzed by one-way ANOVA and using the Tukey post-hoc test. *P* values of < 0.05 being considered statistically significant (***p < 0.001; **p < 0.01; *p <0.05).

The effects of morphine in the embryos are probably mediated by oprm1, the opioid receptor that exhibits highest affinity towards morphine [46]. Thus, in order to establish the role of *oprm1* in the expression of the identified genes in our study, we microinjected specific morpholino (MO) to knock-down (KD)-oprm1 at the one-to-four-cell stage in the yolk (named *oprm1*-MO group; Figure 7), embryos were analyzed when the 24 hpf stage was reached.

After the MO microinjection of oprm1 we did not detect any morphological alteration in the embryos. We found a decrease of the mRNA expression level approximately 95% (data not shown) in the *oprm1* which was similar to that was reported by previous studies of our group [54–56].

To establish that changes observed after MO microinjections were produced by MO action on their target, we also employed two more experimental groups (see Figure 7): embryos injected with standard MO (not targeting any gene in zebrafish, named MO Control group), and embryos with *oprm1*-MO injection plus exposure to morphine (*oprm1*-MO + morphine group).

As shown in Figure 7, our results demonstrated that the gene expression of *grb2*, *acox1*, *dao.1*, *wls*, *grik-l*, *otpb* and *copb2* in the *oprm1* morphants group (*oprm1*-MO) was decreased with respect to the Control group, indicating that oprm1 is important in the expression of these genes (Figure 7A-G). When the *oprm1*-MO group was exposed to morphine (*oprm1*-MO + morphine group), *grb2* expression was recovered and similar values were reached with respect to Control groups, including MO Control group (Figure 7A). In contrast, analyzing the gene expression levels of *acox1*, *grik-l*, *otpb* and *copb2* in the *oprm1*-MO + morphine group, we observed an increase of these genes compared to the *oprm1*-MO group. The comparison of gene expression levels of these four genes in the *oprm1*-MO + morphine group relative to the two Control groups shows that gene expression levels of *grik-l, otpb* and *copb2* were comparable to the Control groups (Figure 7E-G), but in the case of *acox1*, it showed a significant increase as compared to Control groups (Figure 7B). Furthermore, *dao.1* and *wls* expression did not undergo any significant change between *oprm1*-MO + morphine and *oprm1*-MO groups (Figure 7C-D).

Unlike the previous group of genes, the absence of *oprm1*-mRNAs (*oprm1*-MO group) increased the expression of *bnip4*, *camkγ1b* and *sox19b* genes (Figure 7H-J). Thus, silencing gene transcription *oprm1* indicates that it appears to be important in some way in the expression of these three genes. When the *oprm1*-MO group was exposed to morphine (*oprm1*-MO + morphine group), *sox19b* expression was recovered and similar values were reached with respect to the Control groups (Figure 7J). In contrast, *camkγ1b* and *bnip4* expression did not undergo any significant change between *oprm1*-MO + morphine and *oprm1*-MO groups (Figure 7H-I). Finally, *magi1b* and *plg* expression did not present any significant change among all experimental groups (Figure 7K-L).

In conclusion, we found that morphine deregulated (increased or decreased) the expression of *grb2*, *acox1*, *grik-l*, *otpb*, *copb2 and sox19b* through an unknown (not related to oprm1) mechanism; however, it required the expression of *oprm1* to exert its influence on the expression of *dao.1*, *wls*, *bnip4* and *camkγ1b*. In other words, these four genes represent a putative collection of genes whose expression is related to *oprm1* expression.

## Discussion

Most of the published data addressing addictive drugs have focused on adults or neonates in mammalian models [7–19, 57, 58]; little information has been published concerning embryonic development and addiction (e.g. [59] –alcohol-; [10] –morphine-). Nevertheless, the use of morphine by pregnant women, besides affecting them personally, also affects their fetuses [60]. It is known that exposure to morphine may have additional consequences in the mammalian newborn, affecting immune function, neurodevelopment [61] and long term neurobehavioral effects in combination with stress [10, 62].

In the present study, we used zebrafish embryos, since in this model external development can be observed, which is advantageous for the study of the actions of drugs in a specific embryonic stage (see our recent studies with cocaine: [55, 56, 63]).

We described four groups of genes regulated by chronic morphine treatment in zebrafish embryos. Each group contains both, genes already known to be related to morphine activity, and new genes that have not been related to morphine until now. We have also described some genes whose expression is related to *oprm1* expression: (i) Immediate early genes (IEG) and other genes related to transcription; (ii) genes involved in monoaminergic neuromodulation; (iii) genes involved in glutamatergic neurotransmission; and (iv) others genes involved in neuronal function.

### Immediate early genes and other genes related to transcription

Genes previously known are IEGs, such as members of the *fos* and *jun* families, which have important roles in processes such as brain development, learning and long-term neuronal plasticity [64]. Rapid and transient induction of *c-Fos* and *Jun-B* has been described in response to acute morphine administration in different rat brain regions [7]. Our results showed that a duplicate of *junb* was down-regulated (*junbb*, −1.36 FC) as well as their ortholog was up-regulated (*junba*, +1.18 FC). This fact suggests that both genes have different functions, such as reported by Postlethwait *et al*. [65] indicating a subfunctionalization of a gene can occur after a gene duplication event. Additionally, c-*fos* was also up-regulated (+1.15 FC). Other known genes deregulated by morphine in zebrafish embryos and related to transcription are the zinc finger and BTB domain containing 16 (*zbtb16*; +1.44 FC) and the myeloid leukemia factor 1 (*mlf1*; +1.44 FC). For both genes some similar results have been reported in various studies. Thus, z*btb16* was also induced after acute treatment by morphine in mice [12], and this induction was lower after prolonged administration. Furthermore, this gene was found to be positively related to CPP induced by morphine in mouse [66]. Anghel *et al*. [8] showed that the *Mlf1* was also up-regulated by short-term morphine and conversely down-regulated by long-term morphine in the pituitary gland of mice.

We also detected other genes involved in the regulation of transcription that have not been identified in previous studies after morphine treatment, for example, *sox19b* (−1.80 FC), *sox19a* (−1.15 FC), *dbx1b* (−1.36 FC), and *her4*.2 (−1.32 FC) that were also deregulated by amphetamine in zebrafish, like other members of these gene families (*sox9a*, *her15* and *dlx1a*) [27]. With respect to *sox19b*, a gene that we validated by RT-qPCR, belonging to the family of SoxB1 transcription factors (sox1/2/3/19a/19b), its expression is restricted to the developing CNS, and specifically at 24 h of development in zebrafish, it is expressed in the dorsal spinal cord, in the hindbrain, in the tegmentum of the midbrain and in the telecenphalon [67], acting as an important determinant during such anterioposterior regionalization process of the CNS in zebrafish development [68, 69]. Therefore, we can postulate that the down-regulation of *sox19b* produced by morphine exposure in zebrafish embryos could intervene in some way by altering the CNS anterioposterior patterning process.

### Genes involved in monoaminergic neuromodulation

It is widely known that alterations of biogenic amines, neurotransmitters derived by the enzymatic decarboxylation of naturally occurring amino acids (catecholamines, dopamine and norepinephrine, as well as serotonin and acetycholine), by drugs of abuse play an important role in the CNS [70]. In particular, morphine increases dopaminergic neurotransmission in the nucleus accumbens via the activation of dopamine cells in the ventral tegmental area (VTA), an area that possesses a high density of OPRMs (and/or delta opioid receptors, OPRDs), which may mediate reinforcing effects of morphine [71]. This activation mainly results from the disinhibition of inhibitory GABAergic interneurons in the VTA. In zebrafish, dopaminergic and serotonergic systems share similarities with their mammalian counterparts [72, 73]. Although in mammals, dopaminergic neurons have been observed in the diencephalon, telencephalon and mesencephalon; in zebrafish, these neurons have only been detected in the diencephalon and telencephalon [74, 75], and appear during development between 18–22 hpf in zebrafish embryos [76]. In particular, zebrafish A11-type dopaminergic neurons (homologous to mammals), the major far-projecting dopaminergic neurons in this teleost, are located in the ventral diencephalic (hypothalamus) and posterior tuberculum and express specifically the transcription factor Otp [75, 77]. We hypothesized that the dopaminergic neurotransmission during development may be altered by down-regulation of *otpb* gene. In zebrafish, two paralogous *otpa* and *otpb* genes have been previously reported [78] and the requirement for *otpa* and *otpb* function during development of the larval diencephalon is partially redundant. Both genes are essential for the development of specific subsets of diencephalic dopaminergic neurons in zebrafish and mice, of neuroendocrine cells and of specific neurons in the hindbrain in zebrafish [79–83]. Recently, Barreto-Valer *et al*. [63] showed that cocaine also down-regulates both duplicate *otp* genes at 24 hpf zebrafish embryos, and these transcription factors, besides LIM homeobox transcription factor 1 and 2-beta (*lmx1b.1/2*), were important in the expression of tyrosine hydroxylase (*th*), the enzyme responsible for the biosynthesis of dopamine, during the 24 and 48 hpf zebrafish embryonic stages [79].

Another new identified gene, involved also in dopaminergic neurotransmission and down-regulated by exposure to morphine in zebrafish embryos, is *copb2*. This gene encodes the subunit (of 7 subunits) of coatomer complex, which is implicated in a variety of functions ranging from signal transduction, vesicular trafficking and transcriptional regulation to cell cycle control and apoptosis [84]. In particular, *Copb2* regulates the transport of dopaminergic receptor D1 [85]. Thus, one mechanism by which drugs of abuse can regulate the function of dopaminergic receptors is promoting a decrease in the traffic of dopaminergic receptors from the cytoplasm to the cell membrane or vice versa [15]. The trafficking process could be regulated by changes in gene expression of proteins involved in this process, such as Copb2. On the other hand, Kily *et al*. [26] detected that *copb2* was down-regulated by treatment of both nicotine and ethanol in zebrafish; these authors also demonstrated that *copb2* is implied in the development of the notochord.

Concerning genes involved in the serotonergic signaling pathway that in our study are deregulated by chronic morphine treatment, we can mention *bnip4* (also named *bnip3b*), an unknown gene to date in the context of animals treated with morphine. We validated by RT-qPCR the results obtained by microarrays analysis and identified that the expression of this gene is related to *oprm1* expression. In zebrafish, three ortholog genes have been described: *bnip3* and *bnip4*, not induced by hypoxia, and *bnip3a*, which is induced by hypoxia and is much closer to human BNIP3, which is related to cell death/survival [86]. In addition, it is known that rat *Bnip3* gene is differentially regulated during development and induces both apoptosis and autophagy. Besides it is also involved in other biological processes, such as mitochondrial dynamics and intracellular calcium regulation [87]. We can hypothesize that the increase of *bnip4* expression produced after exposure to morphine may be involved in a possible induction of apoptosis/autophagy.

It has interestingly been suggested that mammalian BNIP3 protein can be a candidate for an intrinsic antidepressive effect-related factor and an antistress reaction factor [88, 89]. Enhanced *Bnip3* expression in NG108-15 cells, which possess the serotonin 2C receptor (5-HT2CR) mRNA system, was observed after exposure to Hochu-ekki-to (HET), a (Wakan-yaku) Sino-Japanese traditional drug with antidepressive effects, as well as after exposure to typical antidepressants [88]. In addition, rat brain Bnip3 mRNA expression is enhanced under stress conditions [89]. Thus, both studies speculate that Bnip3 mRNA may be the candidate gene that is controlled by the 5-HT2CR mRNA system, which plays an important role in the action of antidepressants together with serotonin transporters and/or noradrenaline transporters, and also may play a role in the maintenance and/or reformation of synapses in the brain. Therefore, data published up to now [86–89] and our own data suggest that antidepressants in mammals and the treatment with morphine in zebrafish embryos producing an increase in expression of *Bnip3-type* genes may initially seem to indicate an unfavorable pro-apoptotic state of the neuronal cell. In addition, a second conclusion of functional significance of *bnip4* in zebrafish is that the study of this teleost during development may be a good model to confirm that *bnip4* mRNA is a candidate for one of the genes that are controlled by the 5-HT(2C)R mRNA system. The relation between morphine and serotonergic neurotransmission is supported by biochemical studies that demonstrated reciprocal functional interactions of the 5-HT(2A) receptor and the OPRM1 following activation by morphine [90].

### Genes involved in glutamatergic neurotransmission

In addition to monoaminergic neurons, the glutamatergic system plays a critical role in drug dependence and addiction [91]. In relation to this system, we identified groups of genes that are associated with the modulation of glutamate signaling, producing neuroplasticity, and are regulated by chronic morphine treatment, some of which (as *grik-l*, *slc1a4* and *slc1a3a*) have not been described before. Plasticity following chronic drug exposure, including morphine, has been described in the extended amygdala (formed by nucleus accumbens and central amygdala) and, in particular, at the interface between glutamatergic and monoaminergic systems [58, 92].

Within the group of genes down-regulated by exposure to morphine in zebrafish embryos, we found *dao.1*, which we have validated by RT-qPCR and identified as a gene related to *oprm1* expression. This gene encodes a peroxisomal flavoprotein (DAO) that catalyzes the oxidative deamination of neutral and polar D-amino acids as D-Ser (a co-agonist of N-methyl-D-aspartate (NMDA) receptor [93]) to hydrogen peroxide, and is expressed in mammals in the kidneys, liver and almost exclusively within astrocytes in the spinal cord [94–96]. Unlike our study, two papers of Yoshikawa *et al*. [51, 52] showed that acute and chronic treatments with morphine increases the expression of *Dao* and serine racemase (*Sr*), which catalyzes the direct formation of D-Ser from L-Ser, in most parts of rat brain, mainly forebrain. In our study, we detected that both genes were down-regulated by morphine exposure in zebrafish embryos (in the case of *sr*, −1.20 FC). We have found several genes, as for example *dao.1*, chemokine (C-X-C motif) ligand 12a (*cxcl12a*), NADH dehydrogenase (ubiquinone) 1 beta subcomplex 2 (*ndufb2*), phytanoyl-CoA dioxygenase domain containing 1 (*phyhd1*), and carbonic anhydrase II (*ca2*) that were regulated in opposite directions in our study in contrast to other studies; such differences may be due to different morphine exposure areas, tissue (brain) region and/or species.

Interestingly, in rodents, spinal DAO contributes to the development of central sensitization-mediated chronic pain and may be a potential target molecule for the treatment of chronic pain and as an efficacious molecule mediating morphine tolerance [96, 97]. In conclusion, the decrease of *dao.1* expression by treatment with morphine in zebrafish embryos contrary to that obtained with rodents opens a door hitherto unknown in the use of zebrafish as a model to study chronic pain and the effect of morphine in relation to the *dao.1* gene. Future studies will be required to resolve these unknowns, concerning the mechanisms of action of this gene in zebrafish.

Other gene involved in serine metabolism, whose expression was also down-regulated by exposure to morphine in zebrafish embryos, is the transporter of neutral amino acids *slc1a4* (named also ACT; −1.27 FC). SLC1A4 seems to be the main uptake system of L-Ser in neurons [98]. Therefore, the presence of at least three genes differentially expressed after exposure to morphine and involved in serine metabolism implies that morphine can alter this type of metabolism influencing multiple processes involved in this amino acid, such as the regulation of NMDA receptors [99]. Other genes related to the primary excitatory amino acid neurotransmitter glutamate, and closely related to signal transduction, whose expression was altered by morphine treatment in zebrafish embryos were: a kainate-like receptor (*grik-l*); the transporter of glutamate *slc1a3a* (+1.18 FC); glutamate-ammonia ligases 1a and b (*glula*: +1.14 FC and *glulb*: −1.19 FC); and glutamate dehydrogenase 1b (*glud1b*: −1.18 FC). In the case of *grik-l*, we identified by *in silico* studies that Dr.3211.1.A1 probe set target sequence is complementary to deep 3’ untranslation region (UTR) of *si:ch211-251b21.1* mRNA, which encodes a protein similar to mammalian glutamate ionotropic receptors, in particular, a kainate-like receptor [99]. Furthermore, Xu *et al*. [100] demonstrated that si:ch211-251b21.1 as a target gene is regulated by hedgehog signaling during development, and their expression was confined to the dorsal neurons of the spinal cord in wild-type zebrafish embryos at 24 hpf. On the other hand, it is known that morphine causes alterations of gene expression in distinct subunits of postsynaptic glutamatergic receptors, including kainate receptors [101]. Therefore, we suggest that the alteration of gene expression of *grik-l* by exposure to morphine could modify neural development of the spinal cord. Concerning *slc1a3*a gene, it is known that alteration of the expression of high-affinity glutamate transporters, like this gene, has been reported in drug dependence and addiction animal models [102]. In zebrafish, two orthologs for *slc1a3* were found [103]. *slc1a3a* was expressed in glia cells of the larval zebrafish brain similar to the expression of mammalian SLC1A3 (also named GLAST or EAAT1) throughout the CNS [97, 103, 104]. SLC1A3 is critical to maintain the extracellular glutamate concentration in a non-neurotoxic range [97]. Interestingly, in mammals, it has been reported that the expression of SLC1A2 mRNA (or GLT1 or EAAT2), but not SLC1A3, is decreased in the striatum/nucleus accumbens and thalamus of morphine-dependent rats [105]. Therefore, we suggest that zebrafish *slc1a3a*, whose expression was down-regulated in embryos exposed to morphine, could be the key glutamate transporter in the regulation of the glutamate homeostasis in this species, and not *slc1a3b* or *slc1a2*. Finally, we also have observed opposite expression for the two orthologs *glul* genes, suggesting that both genes have different functions, such as reported Postlethwait *et al*. [65]. *glulb* expression is diminished in the same way as mice *Glul* is suppressed by morphine and by other addictive drugs, as methamphetamine, cocaine and alcohol [106].

### Others genes involved in neuronal function

Within the group of genes up-regulated by exposure to morphine in zebrafish embryos, we found *camk1γb*, validated by RT-qPCR and identified as a gene related to *oprm1* expression. This gene is involved in Ca^2+^ signal transduction in neuronal development, such as dendritogenesis and axonogenesis [107, 108]. In particular, CaMKI contributes strongly to Ca^2+^-mediated transcription in neurons through crosstalk with the Ras/extracellular-signal-regulated kinase (Erk) pathway. In cultured hippocampal neurons or acute slices, NMDA-stimulated activation of Erk is predominantly mediated through Ca^2+^/calmodulin-dependent protein kinase kinase (CaMKK)/CaMKI. Furthermore, this pathway appears to be important for dendritic arborization where activity-dependent NMDAR activation of the γ isoform of CaMKI results in MEK/Erk-mediated CREB regulated transcription of Wnt-2 and microRNA132 [108]. Therefore, we hipothetize the overexpression of *camk1γb* produced by morphine exposure in zebrafish embryos may result in pronounced acceleration of axon formation, such as reported by Davare *et al*. [107].

Another gene down-regulated by exposure to morphine and identified as a gene related to *oprm1* expression is the *wls*, a putative orphan G-protein coupled receptor (GPCR) that encodes a OPRM1 interacting protein, and is conserved from worms to human [108]. Juul *et al*. [10] reported that *Wls* was also down-regulated by treatment with morphine in a mouse model of neonatal stress, which suggests that it can promote the inhibition of Wnt secretion. *Wls* is expressed in various brain regions and peripheral tissues in mammals and during zebrafish embryogenesis suggesting that WLS may play an essential role in regulating secretion of multiple Wnts throughout the body and specifically, it might be critical for neuronal development and morphogenesis among other functions [109–112]. In particular, zebrafish *wls* persists at 24 hpf in the spinal cord and in different areas of midbrain, hindbrain, midbrain-hindbrain boundary and ventricular zone. Using antisense morpholinos to KD *wls* mRNA translation in developing zebrafish, Jin *et al*. [110] suggested that *wls* expression was required for brain and ear development during zebrafish embryogenesis. WLS may possibly serve as a substrate underlying the alterations in neuronal structure and synaptic organization characteristic of opioid dependence [113]. Regarding these dependence processes, it should be emphasized that WLS and OPRM1 have been co-localized in somata and in dendritic processes in the murine striatum [113, 114], and that proteins that interact directly with the OPRM1, as WLS, influence their biosynthesis, trafficking and signaling [115], suggesting that these proteins could regulate these types of mechanisms, including signaling and trafficking. Reyes *et al*. [116] hypothesized that when morphine binds with OPRM1, the morphine-enhanced interaction between OPRM1 and WLS causes entrapment of WLS at the cell surface, and WLS is inefficiently internalized. Subsequently, a larger proportion of OPRM1 and WLS are present at the plasma membrane enabling more OPRM1 to be available for activation by morphine. Thus, inhibiting WLS function in mediating Wnt secretion is related to a significant inhibition of Wnt secretion in treatment with morphine. While WLS is inefficiently internalized after morphine treatment, WLS is efficiently internalized in the presence of [D-Ala, N-MePhe, Gly(ol)]-enkephalin (DAMGO) [113, 116] as it is known that happens with morphine. In conclusion, *wls* down-regulate expression by treatment with morphine suggesting a decrease downstream Wnt signaling, affecting neuronal development (structure and synaptic organization) and ear development in particular [109, 110, 112, 113]. Furthermore, we hypothesize that the decrease in *wls* expression could be a direct response mediated by the OPRM1. Two reasons suggest this: (i) our demonstration that *wls* is a gene related to OPRM1 expression, and (ii) a direct interaction at the protein level of WLS and OPRM1. Therefore, both gene expression and protein levels of *wls*/WLS are regulating actions of morphine through OPRM1.

## Conclusions

We present here detailed changes in transcriptome of a critical period of zebrafish development, at 24 hpf, a key stage in the maturation and differentiation of CNS, during chronic treatment of morphine.

Using microarray technology, we identified different functional classes of genes and individual candidates (differentially expressed genes) involved in the mechanisms underlying susceptibility to morphine actions related to CNS development and, in general, the neural function. In particular, we identified 1023 genes whose expression is altered after chronic morphine exposure in zebrafish embryos and, of these, 254 genes had a FC of at least 1.3.

We found morphine-induced changes in gene expression that are specific for the zebrafish and other genes that are similar to mammals. Several morphine-induced genes were exclusively detected in zebrafish embryos, which may be putative targets to analyze, in human models, the problems of addiction and pain. In addition, we demonstrated that some of morphine-induced genes identified in our study might also be related in some way with the opioid system, in particular, the *oprm1* expression, which could open new lines for the treatment of pain and the molecular mechanisms involved in addiction.

We suggest that some of the genes differentially expressed after chronic morphine exposure in zebrafish embryos could produce alterations in neuronal development, in particular, in notochord (in the case of *copb2*), spinal cord (*grik-l*) and other brain regions (*wls* and *dao.1*), CNS patterning processes (*sox19b*), differentiation and dopaminergic neurotransmission (*otpb* and *copb2*). Besides a possible induction of apoptosis and/or autophagy and alteration of serotonergic signaling pathway by the deregulation of *bnip4*, and activation of processes of axonogenesis and dendritogenesis (*camk1γb*) could be present in our experimental model. Finally, the down-regulation of *dao.1* expression by treatment with morphine in zebrafish embryos contrary to that obtained with rodents opens a door hitherto unknown in the use of zebrafish as a model to study chronic pain and the effect of morphine in relation to this gene.

In conclusion, morphine in distinct species can affect common targets and some of these molecular targets may turn out to be central in understanding pain and addiction processes.

## Methods

### Animals

Adult zebrafish (wild-type AB strain) were raised in a cycle of 14 h light: 10 h dark at 26°C in a multi-tank system at our Fish Facilities at the Institute of Neuroscience of Castile & Leon, University of Salamanca. Embryos obtained from natural fertilization were selected at 24 hpf using a Discovery V8 stereomicroscope (Carl Zeiss, Germany), after which fish were raised at 28.5°C and maintained in dishes containing sterile E3 medium (5 mM NaCl, 0.17 mM KCl, 0.33 mM CaCl, 0.33 mM MgSO_4_) in distilled water. Embryos staged according to development in hpf according to Kimmel *et al*. [42].

### Ethics statement

All procedures and experimental protocols were carried out in accordance with the guidelines of the European Communities Council directive of 24 November 1986 (86/609/EEC), current Spanish Legislation (RD 1201/2005, BOE 252/34367-91, 2005), and following the Guide for the Care and Use of Laboratory animals as adapted and promulgated by the US National Institute of Health. All efforts were made to minimize the number of embryos used and their possible suffering.

All experiments were performed at the Institute of Neuroscience of University of Salamanca with the approval of the Animal Care and Ethics Committee of this Institution.

### Microarray study design and drug treatment

Zebrafish embryos were divided into two experimental groups: control embryos and embryos at 5 hpf (end of blastula) exposed to 10 nM morphine and collected at 24 hpf, in order to study the chronic effects of the exposure to drug. Morphine was administered to the embryos in their water environment, i.e., diluted in E3 embryonic medium. Microarray experiments were performed using six replicates for each condition, which contained the RNA of approximately one hundred embryos to minimize the influence of potential individual differences between the animals and technical variation introduced by tissue preparation. We previously reported that a concentration of 10 nM morphine is the highest concentration that can be used without a toxic effect on the embryos, and close to 5% of the morphine diluted in the E3 medium is detected in the embryo [54]. Morphine was acquired from the Spanish Ministry of Health.

### RNA isolation and microarray hybridization

Total RNA was purified using TRIZOL® (Gibco BRL, Gaithersburg, MD, USA) following further RNA purification using an RNeasy Mini Kit for RNA clean-up (Qiagen Sicences, Maryland, USA). RNA quantification and quality was then assessed using Agilent 2100 Bioanalyzer (Agilent Technologies, Palo Alto, CA, USA), to test the integrity of the 18S and 28S rRNA bands, and samples with an RNA integrity number (RIN) > 8.0 were used.

Microarray analysis was performed in the Cancer Research Center (CIC) of Salamanca according to standard procedures. Labelling and hybridizations were performed according to protocols from Affymetrix. Briefly, 100–300 ng of total RNA were amplified and labeled using the WT Sense Target labelling and control reagents kit (Affymetrix Inc., Santa Clara, CA, USA), and then hybridized to GeneChip® Zebrafish Genome Array (Affymetrix). Washing and scanning were performed using *GeneChip* System of Affymetrix (GeneChip Hybridization Oven 640, GeneChip Fluidics Station 450 and GeneChip Scanner 7G).

### Microarray hybridization data analysis: normalization, differential gene expression and ontological analysis

The RMA (Robust Multi-array Analysis) algorithm [117] was used for background correction and normalization of fluorescent hybridization signals of the microarrays, both at internal (intra-microarrays) and comparative (inter-microarrays) levels. This algorithm was selected over others available (MAS5, (Affymetrix 2001); MBEI, a model-based algorithm) [118] because it was deemed to provide the best precision in signal detection to achieve adequate multiple-chip normalization [119], especially in cases of low-level gene expression [117, 120, 121] by producing efficient quantile normalization of the distribution of probe intensities from each array in the context of a complete set of arrays. We used Bioconductor and R as computational tools (http://www.bioconductor.org), to apply RMA to the data set of 12 microarray hybridizations including six different biological replicas corresponding to each of the different experimental groups under study (Control and Morphine).

After quantitation of expression level of each probe set in all microarrays analyzed, the SAM algorithm [122] was used to identify probe sets displaying significant differential expression when comparing the treat samples to it controls. This algorithm performs statistical discrimination analysis using permutations to check the stability of variables fulfilling the ‘alternative hypothesis’. The method calculates the type I error, or number of expected false positives, using the calculation of the False Discovery Rate (FDR) parameter [123]. In this report, genes with an FDR of 10% or less were considered significant.

Further processing included functional analysis and over representation calculations based on Gene Ontology (GO) Annotation Tool and publication data of Database for Annotation, Visualization, and Integrated Discovery was made with GeneSpring GX 7.3 and DAVID Bioinformatics Resources 6.7 (http://david.abcc.ncifcrf.gov/) [124].

### Data access

The data obtained and discussed in this publication have been deposited in NCBI’s Gene Expression Omnibus [125] and are accessible through GEO Series accession number GSE61062 (http://www.ncbi.nlm.nih.gov/geo/query/acc.cgi?acc=GSE61062).

### Quantitative reverse transcription real-time PCR (RT-qPCR)

Total RNA (2 μg), primed with oligo-dT, was reverse-transcribed into cDNA at 37°C for 2 h using the first-strand cDNA synthesis kit (Promega Corporation, Madison, WI, USA) in a 20 μl volume, and stored at −20°C until used, according to manufacturer’s instructions. In all cases, a reverse transcriptase negative control was used for testing genomic DNA contamination.

Quantitative real-time PCR (qPCR) were performed using the SYBR-Green method with a 2× Master Mix (Applied Biosystems). Each reaction contained 10 μL of Master Mix, 0.4 μL of each pair of primers, 3 μL of each cDNA sample in a different serial cDNA quantity for each gene, and MilliQ water up to 20 μl. The amplification reaction took place in an ABI Prism 7000 detection system (Applied Biosystems), with the following conditions: 10 min at 95°C followed by 40 cycles of 15 s at 95°C and 1 min at 60°C depending on each pair of primers. RT-qPCR experiments were performed in replicates of eight and run in triplicate for each gene product examined. The list of used primers is provided in Additional file 6. Zebrafish *β-actin* was used as housekeeping gene.

The comparative Ct method was used for presenting quantitative data [126]. Following the removal of outliers, raw fluorescence data were used to determine the PCR amplification efficiency (E) according to the formula E = [10 ^(−1/slope)^ −1] * 100. All amplifications had an E value of 100 ± 10% the E value close to 100% being an indicator of efficient amplification. The relative gene expression value (FC) for each transcript was calculated according to the equation 2 ^−(ΔCt “condition 1” − ΔCt “condition 2”)^, where “condition 1” corresponds to experimental samples (“treatment with morphine”), “condition 2” to samples of control animals and ΔCt of each “condition” is Ct _“experimental gene”_ − Ct _“endogenous gene”_ [126]. A standard error for each relative gene expression value was calculated as a measure of data variation. Significance from qPCR analysis was determined using a one-way *t*-test for each gene, testing that |FC| > 1 is significant (p < 0.05).

### Mu opioid receptor morpholino microinjection

Antisense MO oligonucleotides used were provided by Gene Tools (LLC Philomath, OR, USA). MO injection was performed according to the methodology developed by Nasevicius and Ekker [127], for which a microinjection and micromanipulation system designed especially for the microinjection of zebrafish embryos was used, coupled to a stereoscope (Stereo Microscope Discovery V8, Zeiss, Göttingen, Germany) and a high-resolution camera that allowed videos and photos to be taken. First, the ideal concentration of MO was determined. Accordingly, several concentrations, looking for small lethal effects and maximum embryonic survival, were microinjected. Thus, the highest concentration with minimum mortality was employed; concentrations of 1 M were used for oprm1 (MOR-MO group). Moreover, parameters of pressure and the appropriate time for microinjecting the volume calculated in each pulse (3 nl of solution) were calibrated. Zebrafish embryo yolks microinjected with MOR-MO and the MO controls were used in the single-cell stage of development. Other embryos, in which no solution was microinjected, were used as a control group. The microinjected and the non-microinjected embryos were maintained under the same conditions: 28.5°C and in the E3 medium (5 mM NaCl, 0.17 mM KCl, 0.33 mM CaCl_2_, 0.33 mM MgSO_4_ in ddH_2_O). To inhibit *oprm1* mRNA translation we used the following MOR-MO sequence: AATGTTGCCAGTGTTTTCCATCATG. The efficacy and specificity of MO employed was demonstrated by Sanchez-Simon *et al*. [54].

## Electronic supplementary material

Additional file 1: **Table listing the probe-sets of genes expressed differentially by chronic morphine treatment in zebrafish embryos.** Significance level for each probe-set are presented as *d-, p*- and *q*-value. The expression changes between Morphine vs. Control are showed as R fold. List of known genes up-, down-, and up-/down-regulated. (XLSX 199 KB)

Additional file 2: **Figure presenting gene ontology analysis of the zebrafish gene collection.** Genes were categorized with the Biological Process domain. Significantly enriched GO terms have a probability lower than 0.01 (*P* value) and include at least three genes. GO terms are shown if they are significantly enriched in at least one of them. Significantly enriched GO terms are indicated as black bars whereas non-significantly enriched terms are displayed as empty bars. Bars represent the number of genes assigned with the corresponding GO term. For more details, see also Additional file 3. (PDF 188 KB)

Additional file 3: **Data file providing the detailed description of Gene Ontology analysis presented in Figure** 2 **and Table** 1 **.** List of probe-sets and gene names classified for each GO category. (XLSX 70 KB)

Additional file 4: **Table listing genes differentially expressed by morphine and included in each functional category presented in Figure** 3 **and Table** 2 **.** The neuronal function category is emphasized in two excel sheet tabs (listing genes and Gene Ontology analysis of genes with neuronal function). (XLSX 98 KB)

Additional file 5: **Table listing genes differentially expressed by morphine validation by our** ***in silico*** **analysis.** (XLSX 34 KB)

Additional file 6: **Table listing the primers used for RT-qPCR.** (PDF 35 KB)

## Abbreviations

CNS: Central nervous system
CPP: Conditioned place preference
Ct: Threshold cycle
FC: Fold change
hpf: Hours post-fertilization
IEG: Immediate early genes
KD: Knocked down
OPRM1: Opioid receptor, mu 1
RT-qPCR: Quantitative reverse transcription real-time PCR
SAM: Significance analysis of microarrays
UTR: Untranslated region.
