# Supplementary figures and images for: Whole-genome expression profile in zebrafish embryos after chronic exposure to morphine: identification of new genes associated with neuronal function and mu opioid receptor expression
Source: BMC Genomics. 2014 Oct 8;15:874. doi: 10.1186/1471-2164-15-874 (PMC4201762; doi:10.1186/1471-2164-15-874)

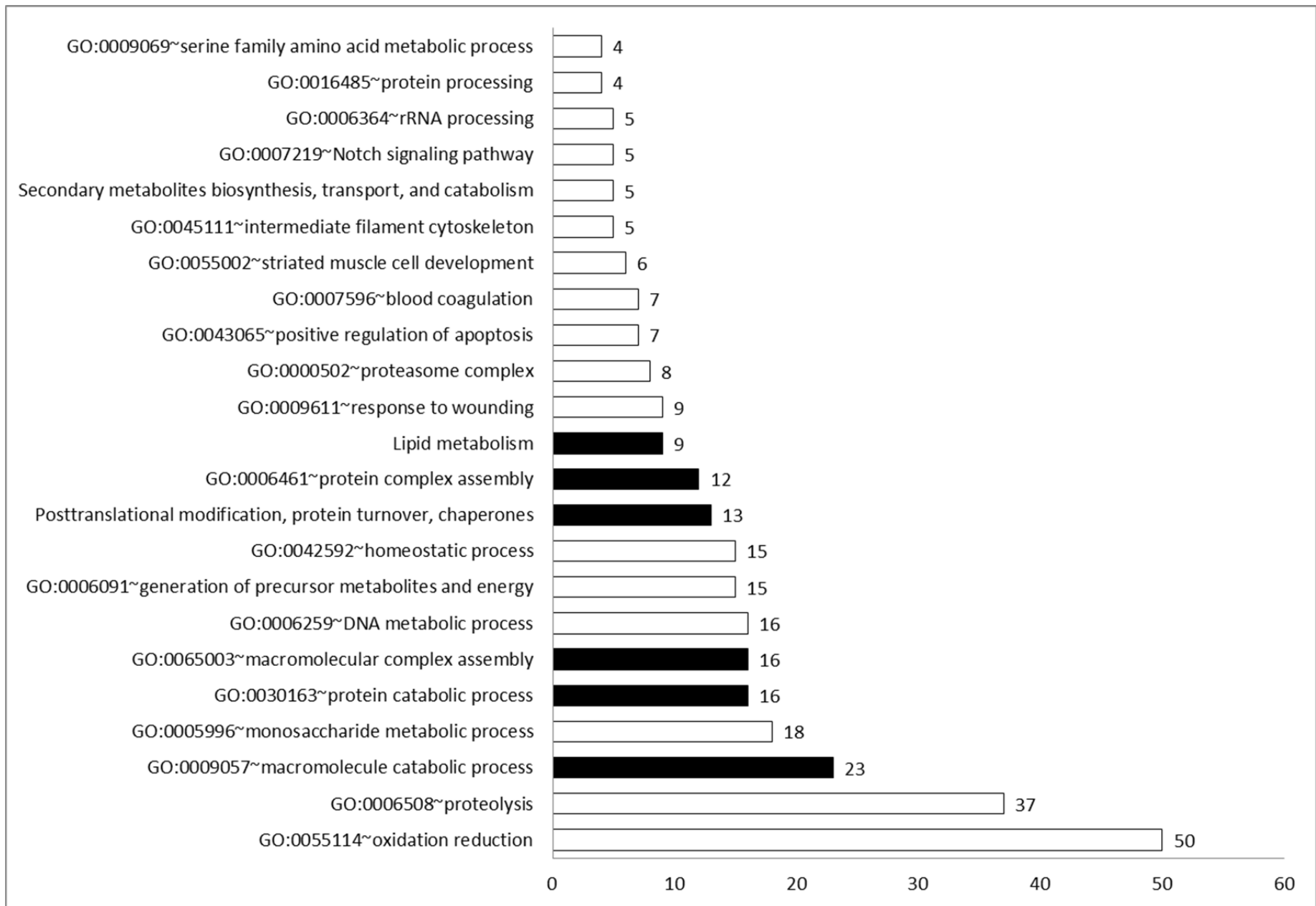

**Additional file 2.**

Supplement: Supplementary file 2 — Additional file 2: Figure presenting gene ontology analysis of the zebrafish gene collection. Genes were categorized with the Biological Process domain. Significantly enriched GO terms have a probability lower than 0.01 (P value) and include at least three genes. GO terms are shown if they are significantly enriched in at least one of them. Significantly enriched GO terms are indicated as black bars whereas non-significantly enriched terms are displayed as empty bars. Bars represent the number of genes assigned with the corresponding GO term. For more details, see also Additional file 3. (PDF 188 KB) [file 12864_2014_6556_MOESM2_ESM.pdf]
