# Supplementary material for: Whole-genome expression profile in zebrafish embryos after chronic exposure to morphine: identification of new genes associated with neuronal function and mu opioid receptor expression
Source: BMC Genomics. 2014 Oct 8;15:874. doi: 10.1186/1471-2164-15-874 (PMC4201762; doi:10.1186/1471-2164-15-874)
Supplement: Supplementary file 6 — Additional file 6: Table listing the primers used for RT-qPCR. (PDF 35 KB) [file 12864_2014_6556_MOESM6_ESM.pdf]

Target protein	Number GenBank*	Primer Forward	cDNA Forward*	Primer Reverse	cDNA Reverse*	Size of products	slope	E*	R^2
**bnip4**	NM_212693	**TGCTTGGATTGGGGATCTGT**	651-670	**GGAGGAAAACAAAAGGCTGTGA**	767-788	138	-3.36	1.98	0.99
**acox1**	NM_001005933	**TTGGACTTTGTGAAAATGGTTCTG**	2330-2353	**TGTGGGTCTTAAGGCATGATTTC**	2405-2427	98	-3.31	2.00	0.96
**dao.1**	NM_001033740	**GCGTCATGAATTGTCCAATGTT**	1252-1273	**GGCATTGCCAGACAGCTACTTT**	1343-1364	113	-3.39	1.96	0.99
**grb2**	NM_213035	**TAACGGCCCCTCACATTCAC**	784-804	**CCTCCATTTCCGTTGGTCTG**	862-882	99	-3.02	2.14	0.98
**sox19b**	NM_131702	**GAACTCCTGCAGGTGGCATT**	2248-2267	**GAACGAATTTTGTGGCAGCA**	2322-2342	95	-3.31	2.00	0.97
**wnl**	NM_213146	**ATTTCCATCCCACTGCGTGT**	1865-1884	**TGCGAGAACGGCTTCTGAAT**	1954-1973	109	-3.50	1.93	0.99
**plg**	NM_201472	**ACAGTTGCCAGGGTGACAGC**	2290-2309	**TCCGGGTTTCATGGCATTAG**	2383-2403	113	-3.45	1.99	0.99
**grik-l**	NM_001144802	**AAGAGATGGAGGACACCTCAGTTT**	1913-1936	**GGACAGAATCTTGGGGATTG**	2009-2029	117	-3.28	2.01	0.99
**magi1b**	NM_001007063	**GGGTTGATTCGCTGCTGAGT**	5306-5328	**GAGAGAGCCGCAACCACAC**	5373-5392	87	-3.16	2.07	0.99
**otpb**	NM_131100	**TGTTGGAGCGATTTTTCAGG**	1517-1537	**TTCCTGTTGATCCCGTTTGAA**	1603-1623	107	-3.12	2.09	0.99
**camk1γb**	NM_200829	**GTGCCGAACACCAAAACTC**	1623-1641	**ACCCCTGCTCCAAATGTGTG**	1704-1723	102	-3.28	2.01	0.99
**copb2**	NM_001001940	**TCAATCTGGATGACGGCTACG**	2852-2872	**GAACCCAGTCCGACTTCAGC**	2969-2988	137	-3.25	2.01	0.99
**b-act**	NM_131031	**ACGACCCAGACATCAGGGAG**	161-180	**CCTCTCTTGCTCTGAGCCTCA**	223-242	82	-3.35	1.99	0.99
**ef1a**	AY422992	**GTACTTCTCAGGCTGACTGTG**	346-366	**ACGATCAGCTGTTTCACTCC**	462-481	136	-3.43	1.96	0.96
**β2mg**	NM_001159768<br>NM_131163	**TTGCCTTCACCCCAGAGAAA**	363-382	**CTGCCCGGTTTGGATTTACA**	450-469	107	-3.45	1.99	0.99

**RPL13a**	NM_212784	**TGGCGGACCGATTCAATAAG**	105-125	**GCCACAATAGCGGAAAGACG**	161-181	77	-3.20	2.05	0.99
